# Pathologic Outcomes following Urethral Diverticulectomy in Women

**DOI:** 10.1155/2014/861940

**Published:** 2014-04-17

**Authors:** Melissa A. Laudano, Asha E. Jamzadeh, Claire Dunphy, Richard K. Lee, Brian D. Robinson, Renuka Tyagi, Steven A. Kaplan, Alexis E. Te, Bilal Chughtai

**Affiliations:** ^1^Department of Urology, Weill Medical College of Cornell University, New York-Presbyterian Hospital, 425 E. 61st Street, 12th Floor, New York, NY 10065, USA; ^2^Department of Pathology & Laboratory Medicine, Weill Medical College of Cornell University, New York-Presbyterian Hospital, New York, NY 10065, USA

## Abstract

*Purpose.* Although most urethral diverticula in women are benign, there is a subset of patients who develop malignant changes. Limited studies report the pathologic findings associated with this relatively rare entity. We describe the clinicopathologic findings of women who underwent urethral diverticulectomy. * Methods.* A consecutive series of 29 women who underwent surgical resection of a urethral diverticulum were identified between 1992 and 2013. Clinical and radiographic data was collected by retrospective review of patient medical records. All pathological slides were rereviewed by a single urologic pathologist. * Results.* Of the 14 women with clinical data, 9 (64%) presented with urgency, 7 (50%) with urinary frequency, 3 (21%) with urinary incontinence, and 3 (21%) with dysuria. Mean diverticular size was 2.3 (±1.4) cm. Although one patient (3%) had invasive adenocarcinoma on final pathology, the remaining 28 cases (97%) demonstrated benign features. The most common findings were inflammation (55%) and nephrogenic adenoma (21%). * Conclusions.* Although most urethral diverticula in women are benign, there is a subset of patients who develop malignancy in association with the diverticulum. In this series, 97% of cases had a benign histology. These findings are important when counseling patients regarding treatment options.

## 1. Introduction

Urethral diverticula are outpouchings of the urethral mucosa into the periurethral tissue or vaginal potential space [1]. Typically, diverticula occur in the middle to distal aspect on the posterior portion of the urethra [2]. Diverticula have a heterogenous morphology, varying in size as well as being either uni- or multilocular [2, 3]. This condition is rare in the general population, with an estimated incidence of 0.6% to 6% [1, 4]. Urethral diverticula are rarely reported in men or children. The age at diagnosis ranges from age 30 to 60 years of age [2].

The etiology of urethral diverticula is generally believed to be acquired rather than being congenital. It is hypothesized that they may result from chronic inflammation associated with recurrent infections such as cystitis [2]. Patients will typically present with a characteristic triad of postvoid dribbling, dysuria, and dyspareunia [3, 5]. Although these are the most common, there is a multitude of other lower urinary tract symptoms that may be associated with urethral diverticula. Acute exacerbations of urethral diverticula usually present with a tender inflamed mass that can be palpated anteriorly at the vaginal wall during pelvic examination [2]. While there are only minimal data comparing diagnostic tests for urethral diverticula, ultrasound is considered a reasonable imaging modality. However, given its higher sensitivity, many now consider MRI to be the gold standard, although it is associated with increased cost [5, 6].

Pathologic findings at the time of diverticulectomy can radically alter the course of treatment. The ability to discuss the probability of benign and malignant findings preoperatively with patients is critical. In this study, we describe the final pathologic characteristics of urethral diverticula in series of cases from our institution.

## 2. Materials and Methods

After Institutional Review Board approval was obtained, 29 consecutive cases of urethral diverticula in women who underwent surgical resection were identified between 1992 and 2013. Clinical and radiographic data were collected by retrospective review of patient medical records. Clinical parameters reviewed, in addition to demographic data, included presenting signs and symptoms, such as dysuria, urinary frequency, urinary incontinence, dyspareunia, and gross hematuria. Radiographic data, such as the diverticular number, size, and location, were also noted. Finally, all pathological slides were retrieved from the archives and rereviewed by a single urologic pathologist at our institution.

## 3. Results

The mean age of women included in this cohort was 44.7 (±14.5) years. Clinical information was available for review in 14 patients. Of these women, 9 (64%) presented with urgency, 7 (50%) with urinary frequency, 3 (21%) with urinary incontinence, and 3 (21%) with dysuria. Eight (57%) women had a history of recurrent urinary tract infections (UTIs). One woman reported dyspareunia (7%), while 3 (21%) experienced urethral discharge. None of the women in our cohort reported gross hematuria.  Table 1 outlines the demographic and clinical data for women in this study.

Of the 29 women, 16 women had radiographic data available for review. Mean diverticular size, measured as the greatest diverticular dimension, was 2.3 (±1.4) cm. Only one woman (6%) had multiple urethral diverticula seen on preoperative magnetic resonance imaging (MRI), while the remaining 15 patients had a single diverticulum seen on either MRI, computerized tomography, or voiding cystourethrogram.  Table 2 outlines the radiographic data in our cohort of women.

All 29 patients underwent a urethral diverticulectomy at our institution and had archived pathologic material available for review. Ten of the diverticula (34%) were lined by squamous epithelium, 9 (31%) by urothelium, 7 (24%) by both urothelium and squamous epithelium, and 1 (3%) by intestinal metaplasia. Two patients (7%) had no identifiable mucosal lining. While the vast majority of the squamous epithelium was of the nonkeratinizing type that is frequently seen in the female urethra, two patients (7%) did have foci of keratinization with associated hyperkeratosis and/or parakeratosis. While one patient (3%) had invasive adenocarcinoma associated with the diverticulum, the remaining 28 cases (97%) demonstrated benign histology. The most common histologic finding was inflammation, present in 16 (55%) of the cases. Twelve of these 16 cases demonstrated chronic inflammation, while the other 4 had an admixture of acute and chronic inflammatory cells. Other common findings included nephrogenic adenoma in 6 (21%) specimens, stromal calcifications in 4 (14%), and ulceration in 3 (10%). A benign leiomyoma was present in association with the diverticulum in 1 patient (3%). Seven women (24%) had no significant histologic changes. The histologic features seen in these resected urethral diverticulectomy specimens are outlined in Table 3.

## 4. Discussion

Diverticula of the female urethra are a challenging problem with regard to diagnosis and surgical reconstruction. Incidence rates range from 0.6% to 6% [1, 4]. When counseling patients with regard to treatment options, one complexity is the possibility of malignancy within the diverticula. The rates of neoplasm within diverticula would be an important piece of information for patients considering surveillance or conservative management. It is also important for the surgeon to consider these rates at the time of diverticulectomy, which may prompt intraoperative frozen section analysis or a wider surgical resection. Given the relatively rare nature of this condition, few small series exist that report pathologic outcomes following urethral diverticulectomy in women.

In this study, we describe the clinicopathologic characteristics of 29 consecutive women who underwent urethral diverticulectomy at our institution from 1992 to 2013. Of women with preoperative clinical data, the most common presenting symptom was urinary urgency (64%), followed by recurrent UTIs (57%) and urinary frequency (50%). Both urinary incontinence and urethral discharge were present in 21% of women. Of women with documented radiographic findings, the majority (94%) had a single diverticulum. The rate of malignancy in our series was extremely low. Only 1 (3%) woman had invasive adenocarcinoma in our series. The most common pathologic finding was inflammation (55%), which was composed of either chronic inflammatory cells (41% of all cases) or mixed acute and chronic inflammatory cells (14% of all cases).

Thomas et al. reported the largest clinicopathologic series of female urethral diverticula collected from 1981 to 2007 [3]. In this series of 90 women, the most common presenting symptom was urinary incontinence (37%), followed by pelvic and urethral pain (35%), and then recurrent UTI (33%). The rates of recurrent UTIs and LUTS were higher in our population, which may be due to true differences in the two cohorts or may simply result from a bias generated by differences in reported patient history. Tsivian et al. retrospectively analyzed clinical, surgical, and pathological data from 22 women who underwent surgery for symptomatic urethral diverticula between 1991 and 2006 [7]. In this group of women, the most common presenting symptom was dysuria (72.2%), followed by dyspareunia (50%) and perineal pain (45.5%). The remaining symptoms included recurrent urinary tract infections (40.9%), purulent discharge (27.3%), incontinence (18.2%), fever (13.6%), urinary outlet obstruction (9.1%), and postvoid dribbling (4.5%). Finally, a third retrospective review by Benjamin et al. reported their series of urethral diverticulum specimens published in 1974 [8]. The primary presenting symptoms were frequency, urgency, and dysuria. Dyspareunia and hematuria were less commonly seen. Similar to our study, the most common presenting symptoms in the cohorts published by Tsivian and Benjamin et al. were urinary complaints, such as dysuria or urgency. All of these studies employed a retrospective collection of clinical data and are therefore subject to reporting bias.

Limited studies reporting on the pathologic findings on diverticulectomy specimens exist in the literature. In the series by Thomas et al., 82 (91%) cases had benign histology [3]. The most common benign histology was chronic inflammation seen in 59 (66%) of cases. This was followed by acute inflammation, squamous metaplasia, and normal urothelium, which were found in 24%, 20%, and 18% of patients, respectively. The authors commented that a significant portion (11%) of diverticula showed nephrogenic adenoma, a benign finding associated with urethral injury or instrumentation. Of note, we found an even higher incidence of nephrogenic adenoma (21%). Similar to the study by Thomas et al., we found that the vast majority of urethral diverticula cases resulted in benign findings. In our series, 28 (97%) patients demonstrated benign findings. The cohort by Tsivian et al. found that the predominant epithelial types were squamous (41.9%), columnar (31.8%), combined squamous and columnar (18.2%), and cuboidal (13.6%). There were signs of inflammation in 77.3% of specimens: 22.7% mild, 22.7% moderate, and 27.3% severe [7]. Although somewhat higher than the rate found in our cohort and the cohort reported by Thomas et al., this study highlights the high rates of inflammation associated with urethral diverticulum.

Malignancy within urethral diverticula remains a rare occurrence, with fewer than 100 cases reported in the literature [9]. The most common malignancy within urethral diverticula is adenocarcinoma, although squamous cell carcinoma is more typically found in the female urethra itself. Thomas et al. reported invasive adenocarcinoma arising in 5/90 (6%) cases and low and high grade dysplasia were found in another 1% and 3% of cases, respectively [3]. We found slightly lower rates of invasive adenocarcinoma in our series at 3%. This is not surprising given its rarity and it may have required a larger patient cohort for detection.

Our series possesses several limitations. The first includes its retrospective nature. This manner of clinical data collection particularly affects the reported signs and symptoms associated with urethral diverticular presentation. In addition, the sample size of our series is relatively small. However, data on pathologic findings within urethral diverticula are lacking from the current literature and, to our knowledge, only one other consecutive case series exists. Finally, we represent the findings collected from a tertiary referral center, so the results may not be generalizable to all practices.

## 5. Conclusions

Limited data exist regarding the rates of various clinicopathologic findings associated with urethral diverticula. Although most urethral diverticula in women are benign, there is a subset of patients who develop malignancy in association with the diverticulum. In this series, 97% of cases had a benign histology. These findings are important when counseling patients regarding management of a urethral diverticulum.

## Figures and Tables

**Table 1:** Clinical data for women undergoing surgical resection of urethral diverticula.

Parameter	Number of patients	Percentage
Age, mean (STD)	44.7 (±14.5)	
LUTS		
Urgency	9	64%
Frequency	7	50%
Dysuria	3	21%
Recurrent UTI	8	57%
Urinary incontinence	3	21%
Urethral discharge	3	21%
Dyspareunia	1	7%
Gross hematuria	0	0%

**Table 2:** Radiographic data for women undergoing surgical resection of urethral diverticula.

Parameter	Number of patients	(%)
Size of diverticula, mean (STD)	2.3 (±1.4) cm	
Number of diverticula		
Single	15	94%
Multiple	1	6%

**Table 3:** Pathologic data for women undergoing surgical resection of urethral diverticula.

	Number of patients (*n* = 29)	%
Type of lining		
Squamous epithelium	10	34%
Urothelium	9	31%
Mixed urothelial and squamous	7	24%
No lining identified	2	7%
Intestinal metaplasia	1	3%
Pathologic findings		
Inflammation	16	55%
Chronic	12	41%
Acute and chronic	4	14%
None/unremarkable	7	24%
Nephrogenic adenoma	6	21%
Stromal calcification	4	14%
Ulceration	3	10%
Leiomyoma	1	3%
Invasive adenocarcinoma	1	3%
